# Assessment of the high risk and unmet need in patients with CAD and type 2 diabetes (ATHENA): US healthcare resource utilization, cost and burden of illness in the Diabetes Collaborative Registry

**DOI:** 10.1002/edm2.133

**Published:** 2020-05-07

**Authors:** Eric Wittbrodt, Narinder Bhalla, Karolina Andersson Sundell, Qi Gao, Liyan Dong, Matthew A. Cavender, Phillip Hunt, Nathan D. Wong, Carl Mellström

**Affiliations:** ^1^ AstraZeneca Gaithersburg MD USA; ^2^ AstraZeneca Gothenburg Mölndal Sweden; ^3^ Baim Institute for Clinical Research Boston MA USA; ^4^ University of North Carolina at Chapel Hill Chapel Hill NC USA; ^5^ University of California Irvine CA USA

**Keywords:** antiplatelet agent, cardiovascular disease prevention, cardiovascular events, epidemiology, type 2 diabetes mellitus

## Abstract

**Background:**

THEMIS (NCT01991795) showed that in patients with type 2 diabetes (T2D) and stable coronary artery disease (CAD) but with no prior myocardial infarction (MI) or stroke, ticagrelor plus acetylsalicylic acid (ASA) decreased the incidence of ischaemic cardiovascular events compared with placebo plus ASA. To complement these findings, we assessed disease burden and healthcare resource utilization (HRU) in US patients with CAD and T2D, but without a prior MI or stroke.

**Methods:**

This observational study used 2013‐2014 data from the Diabetes Collaborative Registry linked to Medicare administrative claims. Two cohorts of patients with T2D were studied: patients at high cardiovascular risk (THEMIS‐like cohort; N = 56 040) and patients at high cardiovascular risk or taking P2Y12 inhibitors (CAD‐T2D cohort; N = 69 790). Outcomes included the composite of all‐cause death, MI and stroke; the individual events from the composite endpoint; HRU; and costs.

**Results:**

Median age was 73.0 years, and median follow‐up was 1.3 years in both cohorts. Event rates of the composite outcome were 16.34 (95% confidence interval: 16.31‐16.37) and 17.64 (17.61‐17.67) per 100 person‐years for the THEMIS‐like and CAD‐T2D cohorts, respectively. The incidence rate of bleeding events was 0.13 events per 100 person‐years in both cohorts. Healthcare costs per patient‐year were USD 8741 and USD 9150 in the THEMIS‐like and CAD‐T2D cohorts, respectively.

**Conclusions:**

Patients in the THEMIS‐like cohort and the broader CAD‐T2D population had similarly substantial cardiovascular event rates and healthcare costs, indicating that patients with CAD and T2D similar to the THEMIS population are at an increased cardiovascular risk.

## Graphical abstract

Disease burden and healthcare resource utilization (HRU) were assessed in patients with coronary artery disease (CAD) and type 2 diabetes (T2D) from a US‐based diabetes registry. Patients similar to those enrolled in THEMIS have substantial cardiovascular event rates and healthcare costs, but low bleeding risk. Findings were similar in a broader CAD‐T2D population, indicating that the THEMIS population reflects high‐risk patients with CAD and T2D in clinical practice.

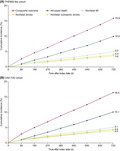

## 1 INTRODUCTION

Cardiovascular (CV) disease is a major cause of morbidity and mortality in patients with type 2 diabetes (T2D), and reduction of CV risk is an important goal of treatment.^1^, ^2^, ^3^ Long‐term (up to 12 months) dual antiplatelet therapy, comprising acetylsalicylic acid (ASA) and a P2Y12 inhibitor (clopidogrel, prasugrel or ticagrelor), is widely used to prevent recurrent ischaemic events in patients with T2D and acute coronary syndrome.^3^, ^4^, ^5^ However, although the magnitude of the independent CV risk conferred by the presence of T2D in the absence of prior ischaemic events has been documented,^1^, ^6^ evidence for a benefit from long‐term use of dual antiplatelet regimens in patients with T2D who have established coronary artery disease (CAD) but who have not experienced a myocardial infarction (MI) or stroke has been inconclusive. Consequently, current clinical guidelines differ in their recommendations for the use of antiplatelet therapy in these patients.^3^, ^7^, ^8^, ^9^, ^10^

The Effect of Ticagrelor on Health Outcomes in Diabetes Mellitus Patients Intervention Study (THEMIS) was a large, randomized, placebo‐controlled trial (ClinicalTrials.gov identifier: NCT01991795) designed to evaluate the efficacy and safety of ticagrelor added to background ASA therapy for the prevention of major CV events in patients with T2D and established CAD but without a history of MI or stroke.^11^, ^12^ THEMIS (N = 19 220) showed that over 36 months of follow‐up the incidence of ischaemic CV events was lower in the ticagrelor group than in the placebo group (hazard ratio [HR]: 0.90, 95% confidence interval [CI]: 0.81‐0.99, *P* = .04),^12^ with greater benefit observed in the predefined subgroup of patients with a history of percutaneous coronary intervention (PCI; HR: 0.85, 95% CI: 0.74‐0.97).^13^

The present analysis, Assessment of The High Risk and Unmet Need in Patients with CAD and Type 2 Diabetes (ATHENA), was designed to complement the clinical data from THEMIS, by providing real‐world insights into the burden of disease in patients with CAD and T2D but without a history of MI or stroke. ATHENA is an observational study, which aimed to assess and describe the following: clinical outcomes including the composite outcome of all‐cause death, nonfatal MI and nonfatal stroke; healthcare resource utilization (HRU); and costs associated with CAD, in two overlapping US populations of patients with T2D (those who would have been eligible for THEMIS and a broader population of patients with CAD and T2D). The study used data from the Diabetes Collaborative Registry (DCR), a US‐based registry of patients with T2D, linked to the Centers for Medicare and Medicaid Services (CMS) administrative claims database.

## 2 METHODS

### 2.1 Data sources and study population

The DCR is a real‐world, quality‐oriented registry led by the American College of Cardiology in partnership with the American Diabetes Association, the American College of Physicians, the American Association of Clinical Endocrinologists, and the Joslin Diabetes Center.^14^ The registry collects real‐world data from a diverse range of care providers, including primary care physicians, endocrinologists and cardiologists. Patients eligible for enrolment in the DCR include those with a diagnosis of diabetes identified through International Classification of Diseases 9th/10th Revision (ICD‐9/10) diagnostic codes. As of 31 March 2016, the DCR included data from 1 029 807 patients across 374 sites and 5114 providers. General practice (including internal medicine, primary care or family practices), cardiology, endocrinology and obstetrics/gynaecology practices were self‐identified in 50.1%, 74.9%, 2.1% and 9.4% of sites, respectively; sites could contain practices with more than one specialty. DCR participation requires no data collection beyond that of routine clinical care and poses no additional risks to clinical providers or their patients; therefore, a waiver of written informed consent and authorization for this study were granted by Chesapeake Research Review, Inc.

The CMS collects data from patients who are enrolled in Medicare or Medicaid healthcare plans in the USA. Available data include records of clinical services used by enrolees, including source of care, dates of admission and discharge, and diagnosis and procedure codes. CMS data can be linked with other databases and registries to create comprehensive data sets.^15^

**FIGURE 1:**
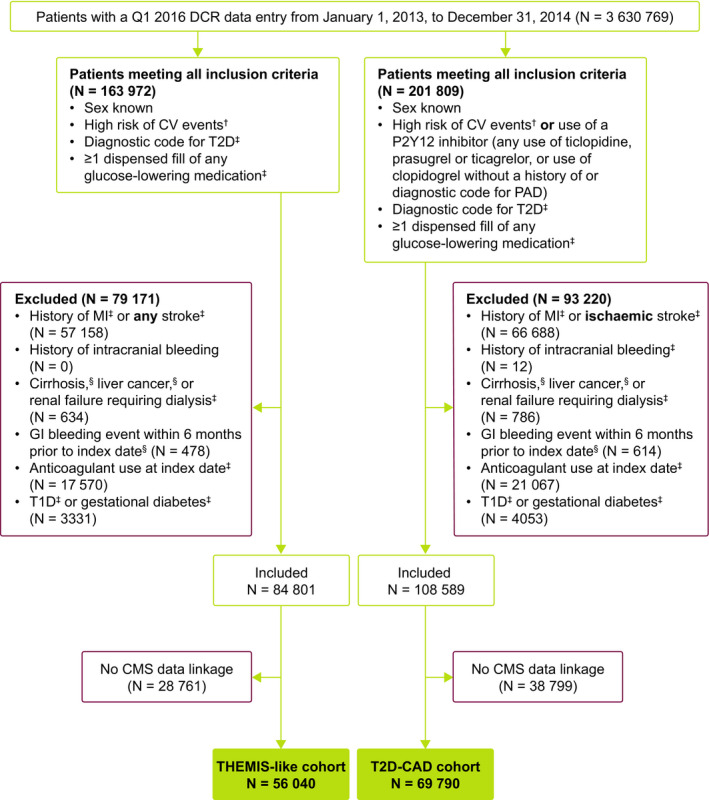
Flow chart showing selection of THEMIS‐like and CAD‐T2D cohorts. For each exclusion criterion, the number of patients is reported on a hierarchical basis (ie exclusion criteria are mutually exclusive). ^†^Defined as having prior PCI, prior CABG or angiographic evidence of ≥50% lumen stenosis of at least one coronary artery (defined by the presence of a code for angina or the Canadian Cardiovascular Society class field [I, II, III, IV] completed in the DCR, without PCI or CABG). ^‡^DCR data. ^§^CMS data. CABG, coronary artery bypass graft; CAD, coronary artery disease; CMS, Centers for Medicare and Medicaid Services; CV, cardiovascular; DCR, Diabetes Collaborative Registry; GI, gastrointestinal; MI, myocardial infarction; PAD, peripheral artery disease; PCI, percutaneous coronary intervention; T1D, type 1 diabetes; T2D, type 2 diabetes; THEMIS, Effect of Ticagrelor on Health Outcomes in Diabetes Mellitus Patients Intervention Study

Selection of the study cohorts is shown in Figure 1. Adults who were seen in a DCR‐participating practice during the time period for which linked CMS administrative claims data were available (1 January 2013 to 31 December 2014), and whose data could be linked with CMS administrative claims data, were eligible for inclusion. Patients were also required to have a diagnostic code for T2D in the DCR and have at least one dispensed prescription for any glucose‐lowering medication.

Two cohorts were defined within the overall set of patients who met these eligibility criteria: THEMIS‐like and T2D‐CAD. The THEMIS‐like cohort (N = 56 040) included patients with a high risk of CV events, defined as having a prior PCI or prior coronary artery bypass graft (CABG), or the presence of a code for angina (ICD‐10‐CM I20.8 [angina pectoris, other] or I20.9 [angina pectoris, unspecified]) or the Canadian Cardiovascular Society class field (I, II, III, IV) without having undergone PCI or CABG (both were used as surrogates for angiographic evidence of ≥50% lumen stenosis of at least one coronary artery). Patients were excluded if they had a history of MI or any stroke (with the exception of transient ischaemic attack [TIA]), a history of intracranial bleeding, cirrhosis, liver cancer, renal failure requiring dialysis or a gastrointestinal bleeding event within 6 months prior to the index date; patients were also excluded if they were taking anticoagulants at the index date.

The CAD‐T2D cohort (N = 69 790) included patients with either a high risk of CV events (defined as for the THEMIS‐like cohort) or patients who were receiving one or more P2Y12 inhibitors (defined as any use of ticlopidine, prasugrel, ticagrelor or clopidogrel; patients using clopidogrel with a diagnosis code for peripheral artery disease but no diagnosis code for angina, PCI or CABG were excluded). Exclusion criteria were the same as for the THEMIS‐like cohort, with the exception that, regarding stroke, only patients with ischaemic stroke (with the exception of TIA) were excluded. As a consequence of these different criteria, the CAD‐T2D cohort consisted of a broader population of patients with T2D than the THEMIS‐like cohort, due to the inclusion of patients with a history of nonischaemic (haemorrhagic) stroke, as well as patients with baseline use of P2Y12 inhibitors.

### 2.2 Analysis

The study index date was defined as the earliest date on which a participant satisfied all study inclusion criteria for either cohort on or after 1 January 2013. Patients were followed up until disenrolment due to death or until the end of the evaluation period (31 December 2014). Demographic and clinical characteristics, including information on medications and laboratory test results, were extracted for patients in each cohort on the index date and are presented descriptively.

Selected clinical outcomes of interest that occurred during follow‐up, including a composite of death, nonfatal MI and nonfatal stroke, and the individual component events, were identified by diagnostic codes (ICD‐9) in CMS claims data. The event rates of all clinical outcomes of interest and 95% CIs were estimated and calculated as the total number of events (incident and recurrent events) divided by the total follow‐up time across patients (sum of the periods of time from index date to death or end of the evaluation period, whichever occurred first) and expressed as events per 100 person‐years. Recurrent events were defined as events that occurred at least 30 days after the incident event. There was no minimum time required between recurrent events. Incidence rates were calculated as the total number of patients with at least one event of interest, divided by the total follow‐up time free from the event of interest across patients. Kaplan‐Meier estimates of the cumulative incidence of clinical outcomes at the end of the 2‐year follow‐up period were determined for the composite outcome and the component events (death, MI, and stroke) for the THEMIS‐like and CAD‐T2D cohorts and in prespecified patient subgroups.

Data relating to HRU, costs and persistence with selected secondary prevention medications (P2Y12 inhibitors, statins, angiotensin‐converting enzyme [ACE] inhibitors, angiotensin receptor blockers [ARBs] and β‐blockers) were analysed from linked CMS data during the 2‐year follow‐up period. Persistence was defined as continuation of a medication class over the study period without more than a 60‐day gap in medication supply after the last fill, as determined by the date and days’ supply dispensed. The denominator for calculations included patients with at least one claim for a particular medication class at baseline. Costs were calculated for the overall study period and by person‐year by dividing the overall costs by the mean duration of follow‐up in each cohort.

## 3 RESULTS

### 3.1 Characteristics of the study cohorts

The THEMIS‐like and CAD‐T2D cohorts comprised 56 040 and 69 790 DCR patients with evaluable data who met the study inclusion criteria, respectively. Demographic and clinical characteristics of patients in the two cohorts were not markedly different (Table 1). Men constituted 62.9% and 61.4% of the THEMIS‐like and CAD‐T2D cohorts, respectively, and the median age in both cohorts was 73.0 years (interquartile range [IQR]: 68.0‐78.0). The proportion of patients with a history of PCI or CABG was 73.0% and 57.7% in the THEMIS‐like and CAD‐T2D cohorts, respectively. More than 90% of patients in both cohorts were receiving an oral antiplatelet (OAP) agent. In total, 85.7% and 83.4% of patients in the THEMIS‐like and CAD‐T2D cohorts, respectively, were taking ASA (as assessed using data from the DCR because ASA use was not available in claims data); 31.9% and 38.2% of patients in the THEMIS‐like and CAD‐T2D cohorts, respectively, were receiving dual antiplatelet therapy.

**TABLE 1:** Patient characteristics at baseline

	THEMIS‐like cohort (N = 56 040)	CAD‐T2D cohort (N = 69 790)
Age, y, median (IQR)	73.0 (68.0‐78.0)	73.0 (68.0‐78.0)
Male, n (%)	35 274 (62.9)	42 882 (61.4)
Ethnicity, n (%)		
White	36 961 (66.0)	45 915 (65.8)
Black	3092 (5.5)	4245 (6.1)
Other	221 (0.4)	293 (0.4)
Missing	15 766 (28.1)	19 337 (27.7)
Height, cm, median (IQR)	170.2 (162.6, 177.8)	170.2 (162.6, 177.8)
Missing, n (%)	4064 (7.3)	5917 (8.5)
Weight, kg, median (IQR)	89.1 (77.3, 102.7)	89.1 (76.8, 102.7)
Missing, n (%)	5305 (9.5)	7765 (11.1)
DBP, mmHg, median (IQR)	70.0 (64.0, 80.0)	70.0 (64.0, 80.0)
Missing, n (%)	2414 (4.3)	3432 (4.9)
SBP, mmHg, median (IQR)	130.0 (120.0, 142.0)	130.0 (120.0, 142.0)
Missing, n (%)	2346 (4.2)	3354 (4.8)
Baseline CV comorbidities, n (%)		
History of heart failure	12 937 (23.1)	15 375 (22.0)
History of AF/flutter (not on anticoagulation medication)	6726 (12.0)	8122 (11.6)
History of stable angina	24 923 (44.5)	24 296 (34.8)
History of peripheral artery disease	11 742 (21.0)	12 252 (17.6)
History of hypertension	49 859 (89.0)	61 643 (88.3)
History of dyslipidaemia	50 078 (89.4)	61 212 (87.7)
History of PCI or CABG	40 886 (73.0)	40 265 (57.7)
Multi‐vessel disease	21 784 (38.9)	21 546 (30.9)
Diabetes complications, n (%)		
History of diabetic neuropathy	623 (1.1)	780 (1.1)
History of diabetic retinopathy	71 (0.1)	100 (0.1)
Metabolic syndrome^a^	11 504/14 680 (78.4)	13 994/17 808 (78.6)
Concomitant medications, n (%)		
OAP^b^	51 156 (91.3)	64 518 (92.4)
ASA	48 041 (85.7)	58 199 (83.4)
Dual antiplatelet therapy^c^	17 872 (31.9)	26 641 (38.2)
Nitrate therapy	4505 (8.0)	5594 (8.0)
ACE inhibitor	28 096 (50.1)	34 838 (49.9)
ARB	16 761 (29.9)	21 069 (30.2)
β‐blocker	44 363 (79.2)	55 002 (78.8)
Calcium channel blocker	19 274 (34.4)	24 473 (35.1)
Diuretic	19 541 (34.9)	24 661 (35.3)
Lipid‐lowering medication	47 185 (84.2)	57 425 (82.3)

Note

Data are mean (SD) unless indicated.

Abbreviations: ACE, angiotensin‐converting enzyme; AF, atrial fibrillation; ARB, angiotensin receptor blocker; ASA, acetylsalicylic acid; BMI, body mass index; CABG, coronary artery bypass graft; CAD, coronary artery disease; CV, cardiovascular; DBP, diastolic blood pressure; HbA_1c_, glycated haemoglobin; HDL‐C, high‐density lipoprotein cholesterol; IQR, interquartile range; LDL‐C, low‐density lipoprotein cholesterol; OAP, oral antiplatelet; PCI, percutaneous coronary intervention; SBP, systolic blood pressure; SD, standard deviation; T2D, type 2 diabetes; THEMIS, Effect of Ticagrelor on Health Outcomes in Diabetes Mellitus Patients Intervention Study.

^a^ Defined as fulfilling three of the following criteria: obesity (BMI >30 kg/m^2^), hypertension (blood pressure ≥130/85 mm Hg), dysglycaemia (HbA_1c_ ≥5.7%), elevated triglyceride level (≥150 mg/dL) or low HDL‐C level (<40 mg/dL if male or <50 mg/dL if female).

^b^ Includes ASA and ASA/dipyridamole (Aggrenox®).

^c^ Defined as ASA and an OAP (clopidogrel, ticlopidine, prasugrel or ticagrelor).

### 3.2 Clinical events

The median duration of follow‐up was 484.0 days (IQR: 272.0‐630.0) for the THEMIS‐like cohort and 471.0 days (IQR: 259.0‐625.0) for the CAD‐T2D cohort. Incidence rates of the composite outcomes were 8.73 (95% CI: 7.46‐8.91) and 9.29 (95% CI: 9.02‐9.44) events per 100 person‐years in the THEMIS‐like and CAD‐T2D cohorts, respectively. The corresponding event rates (first and recurrent events) were 16.34 (95% CI: 16.31‐16.37) and 17.64 (95% CI: 17.61‐17.67) events per 100 person‐years in the THEMIS‐like and CAD‐T2D cohorts, respectively (Table 2), and almost 20% of the patients in each cohort experienced recurrent events. Each component of the composite outcome (nonfatal MI, nonfatal stroke and all‐cause death) contributed similarly to the composite event rate in both cohorts. Between 33.2% and 36.2% of the patients in both cohorts who experienced nonfatal MI, nonfatal stroke or nonfatal ischaemic stroke had a recurrent event (Table 2).

**TABLE 2:** Clinical event and incidence rates

	THEMIS‐like cohort (N = 56 040)	CAD‐T2D cohort (N = 69 790)
Composite outcome^a^		
All events, n	10 775	14 177
Recurrent events, n^b^	2612	3420
Patients with recurrent events, n (%)	1098/5755 (19.1)	1431/7464 (19.2)
Incidence rate per 100 person‐years (95% CI)	8.73 (8.46‐8.91)	9.29 (9.02‐9.44)
Event rate per 100 person‐years^c^ (95% CI)	16.34 (16.31‐16.37)	17.64 (17.61‐17.67)
All‐cause death		
All events, n	3387	4274
Recurrent events, n^b^	–	–
Patients with recurrent events, n (%)	–	–
Incidence rate per 100 person‐years (95% CI)	4.98 (4.81‐5.15)	5.14 (4.99‐5.30)
Nonfatal MI		
All events, n	3481	4494
Recurrent events, n^b^	1169	1515
Patients with recurrent events, n (%)	594/1790 (33.2)	766/2307 (33.2)
Incidence rate per 100 person‐years (95% CI)	2.68 (2.55‐2.80)	2.83 (2.70‐2.93)
Event rate per 100 person‐years^c^ (95% CI)	5.21 (5.19‐5.23)	5.50 (5.49‐5.52)
Nonfatal stroke		
All events, n	3907	5409
Recurrent events, n^b^	1443	1905
Patients with recurrent events, n (%)	526/1453 (36.2)	695/2018 (34.4)
Incidence rate per 100 person‐years (95% CI)	2.17 (2.03‐2.25)	2.47 (2.32‐2.53)
Event rate per 100 person‐years^c^ (95% CI)	5.83 (5.81‐5.85)	6.62 (6.60‐6.63)
Nonfatal ischaemic stroke		
All events, n	3616	5017
Recurrent events, n^b^	1338	1779
Patients with recurrent events, n (%)	479/1330 (36.0)	638/1864 (34.2)
Incidence rate per 100 person‐years (95% CI)	1.98 (1.85‐2.06)	2.28 (2.13‐2.34)
Event rate per 100 person‐years^c^ (95% CI)	5.39 (5.37‐5.41)	6.13 (6.11‐6.15)
Peripheral artery disease		
Incidence rate per 100 person‐years (95% CI)	11.73 (11.46‐12.00)	11.53 (11.29‐11.78)
Hospitalization for heart failure		
Number of events, n (%)	1776 (3.2)	2308 (3.3)
Total number of hospitalizations, median (IQR)	1.0 (1.0, 2.0)	1.0 (1.0, 2.0)
Median length of stay, days (IQR)	3.0 (2.0, 6.0)	3.0 (2.0, 5.7)
Incidence rate per 100 person‐years (95% CI)	2.66 (2.54‐2.79)	2.83 (2.72‐2.95)
Bleeding events, incidence rate per 100 person‐years^d^		
2013 annual incidence rate (95% CI)	0.13 (0.09‐0.18)	0.13 (0.09‐0.18)
2014 annual incidence rate (95% CI)	0.09 (0.07‐0.11)	0.09 (0.07‐0.11)
2‐year incidence rate (95% CI)	0.13 (0.10‐0.16)	0.13 (0.11‐0.16)

Abbreviations: CAD, coronary artery disease; CI, confidence interval; IQR, interquartile range; MI, myocardial infarction; T2D, type 2 diabetes; THEMIS, Effect of Ticagrelor on Health Outcomes in Diabetes Mellitus Patients Intervention Study.

^a^ Nonfatal MI, nonfatal stroke or all‐cause death.

^b^ Recurrent events were defined as events that occurred at least 30 days after the incident event and numbers of patients with recurrent events are expressed as a percentage of the number of patients who experienced at least one event.

^c^ A normal approximation of the estimate of rates based on all events (including recurrent events) was used.

^d^ Nonfatal, nontrauma‐related bleeding events that required an emergency department visit or hospitalization. Incidence rates include recurrent events.

The incidence rate of hospitalization for heart failure was similar in both the THEMIS‐like and CAD‐T2D cohorts (2.66 [95% CI: 2.54‐2.79] and 2.83 [95% CI: 2.72‐2.95] events per 100 person‐years, respectively), as was the incidence rate of peripheral artery disease (11.73 [95% CI: 11.46‐12.00] and 11.53 [95% CI: 11.29‐11.78] events per 100 person‐years, respectively). The incidence rate of bleeding events was low compared with other clinical outcomes in both the THEMIS‐like and CAD‐T2D cohorts (0.13 [95% CI: 0.10‐0.16] and 0.13 [95% CI: 0.11‐0.16] events per 100 person‐years, respectively).

**FIGURE 2:**
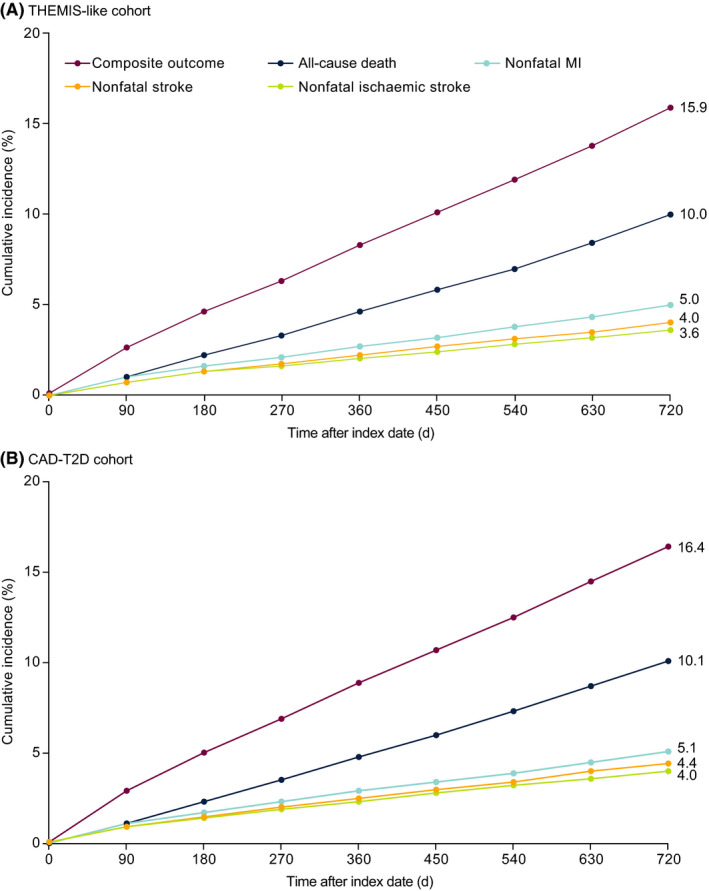
Kaplan‐Meier plots showing the cumulative incidence of the composite outcome (all‐cause death, nonfatal MI and nonfatal stroke), all‐cause death, nonfatal MI, nonfatal stroke and nonfatal ischaemic stroke during follow‐up in (A) the THEMIS‐like cohort and (B) the CAD‐T2D cohort. CAD, coronary artery disease; MI, myocardial infarction; T2D, type 2 diabetes; THEMIS, Effect of Ticagrelor on Health Outcomes in Diabetes Mellitus Patients Intervention Study

Kaplan‐Meier plots showing cumulative incidence of the composite outcome and the individual components over 2 years of follow‐up are shown in Figure 2; the raw data are presented in Table S1. After 360 days of follow‐up, the cumulative incidence of the composite outcome was 8.3% and 8.9% in the THEMIS‐like and CAD‐T2D cohorts, respectively (Table S1). In both cohorts, the incidence of the composite outcome and each component event was higher in patients older than 75 years than in those aged 65‐75 years, and this was also the case in patients with multi‐vessel rather than single‐vessel disease (Table 3). In both cohorts, the incidence of the composite outcome was higher in patients with a history of PCI or CABG than in patients without a history of these interventions, although the difference was more pronounced in the THEMIS‐like cohort than in the CAD‐T2D cohort.

**TABLE 3:** Subgroup analysis: cumulative incidence (95% CI) of CV outcomes at the end of 2 years of follow‐up

Age, years	THEMIS‐like cohort (N = 56 040)		CAD‐T2D cohort (N = 69 790)	
	65‐75	>75	65‐75	>75
N	32 510	20 612	40 402	25 833
Composite outcome^a^	12.4 (11.8‐13.0)	22.5 (21.5‐23.4)	13.0 (12.4‐13.5)	23.0 (22.2‐23.8)
All‐cause death	7.0 (6.5‐7.5)	15.6 (14.8‐16.5)	7.0 (6.6‐7.5)	15.9 (15.1‐16.6)
Nonfatal MI	4.5 (4.1‐4.9)	6.2 (5.7‐6.7)	4.6 (4.3‐5.0)	6.3 (5.8‐6.7)
Nonfatal stroke	3.2 (2.8‐3.5)	5.7 (5.2‐6.2)	3.5 (3.2‐3.8)	6.2 (5.7‐6.7)
Nonfatal ischaemic stroke	2.8 (2.6‐3.0)	5.1 (4.6‐5.6)	3.2 (2.9‐3.4)	5.6 (5.2‐6.1)

Prior PCI or CABG	Yes	No	Yes	No
N	40 886	15 154	40 265	29 525
Composite outcome^a^	16.8 (16.2‐17.4)	12.7 (11.8‐13.5)	16.8 (16.2‐17.4)	15.7 (15.0‐16.3)
All‐cause death	10.7 (10.2‐11.2)	7.6 (6.9‐8.3)	10.7 (10.2‐11.3)	9.0 (8.5‐9.5)
Nonfatal MI	5.2 (4.9‐5.6)	4.2 (3.7‐4.7)	5.3 (4.9‐5.6)	4.9 (4.5‐5.3)
Nonfatal stroke	4.2 (3.9‐4.5)	3.5 (3.0‐3.9)	4.1 (3.8‐4.4)	4.8 (4.4‐5.2)
Nonfatal ischaemic stroke	3.7 (3.5‐4.0)	3.2 (2.7‐3.6)	3.7 (3.4‐3.9)	4.5 (4.1‐4.9)

Vessel disease	Single vessel	Multi‐vessel	Single vessel	Multi‐vessel
N	19 102	21 784	18 719	21 546
Composite outcome^a^	16.0 (15.1‐16.8)	17.6 (16.8‐18.4)	16.0 (15.1‐16.9)	17.6 (16.8‐18.4)
All‐cause death	10.1 (9.4‐10.9)	11.2 (10.6‐11.9)	10.2 (9.4‐11.0)	11.2 (10.5‐11.9)
Nonfatal MI	4.9 (4.4‐5.3)	5.6 (5.0‐6.1)	4.9 (4.5‐5.3)	5.6 (5.0‐6.1)
Nonfatal stroke	4.0 (3.6‐4.5)	4.3 (3.9‐4.8)	3.9 (3.5‐4.4)	4.3 (3.9‐4.7)
Nonfatal ischaemic stroke	3.5 (3.2‐3.9)	3.9 (3.5‐4.3)	3.5 (3.1‐3.8)	3.9 (3.5‐4.2)

Note

Cumulative incidence was calculated using Kaplan‐Meier estimates.

Abbreviations: CABG, coronary artery bypass graft; CAD, coronary artery disease; CI, confidence interval; CV, cardiovascular; MI, myocardial infarction; PCI, percutaneous coronary intervention; T2D, type 2 diabetes; THEMIS, Effect of Ticagrelor on Health Outcomes in Diabetes Mellitus Patients Intervention Study.

^a^ Nonfatal MI, nonfatal stroke or all‐cause death.

### 3.3 Costs and medication persistence

Mean overall total costs during follow‐up for patients who made a claim were USD 15 329 (N = 37 341) and USD 15 476 (N = 46 928) in the THEMIS‐like and CAD‐T2D cohorts, respectively (Table 4). Inpatient total costs were particularly high, with means of USD 21 545 (N = 12 703) and USD 21 770 (N = 16 080) in the THEMIS‐like and CAD‐T2D cohorts, respectively. Across all patients (with or without claims), the costs per person‐year were USD 8741 for the THEMIS‐like cohort and USD 9150 for the CAD‐T2D cohort. Medication persistence during the follow‐up period was similar between cohorts and above 80% for all drug classes assessed. Persistence with P2Y12 inhibitors was greatest in both cohorts (THEMIS‐like: 85.4%; T2D‐CAD: 85.8%). Persistence with statins was lowest in both cohorts (THEMIS‐like: 80.2%; T2D‐CAD: 80.4%).

**TABLE 4:** Costs and medication persistence over 2 years of follow‐up

	THEMIS‐like cohort (N = 56 040)	CAD‐T2D cohort (N = 69 790)
Treatment costs per patient over the study period, USD,^a^ mean (SD) and median (IQR)		
Overall total costs	N = 37 341 15 329 (24 583) 6791 (1823‐18 580)	N = 46 928 15 476 (25 042) 6774 (1813‐18 705)
Inpatient total costs	N = 12 703 21 545 (27 768) 12 475 (6036‐26 786)	N = 16 080 21 770 (28 206) 12 611 (6052‐27 035)
Outpatient total costs	N = 32 716 4323 (8527) 1588 (477‐4173)	N = 41 068 4309 (8519) 1572 (473‐4127)
Outpatient pharmacy costs	N = 26 334 5972 (9276) 3403 (1199‐7470)	N = 33 245 5993 (9553) 3376 (1193‐7464)
Overall total CV‐related costs^b^	N = 23 295 5903 (13 241) 742 (151‐6121)	N = 29 188 6020 (13 709) 745 (153‐6199)
Inpatient total CV‐related costs	N = 6070 16 524 (19 851) 10 729 (5569‐21 256)	N = 7675 16 862 (20 717) 10 832 (5618‐21 554)
Outpatient total CV‐related costs	N = 20 862 1615 (4054) 324 (107‐1166)	N = 26 068 1610 (4058) 324 (106‐1164)
Outpatient pharmacy CV‐related costs	N = 1928 1824 (1160) 1662 (789‐2758)	N = 2402 1799 (1166) 1641 (726‐2719)
Costs per person‐year, USD^c^		
Overall total costs	8741	9150
Inpatient total costs	3824	3954
Outpatient total costs	1935	2002
Outpatient pharmacy costs	3068	3194
Overall total CV‐related costs^b^	1897	2003
Inpatient total CV‐related costs	1370	1461
Outpatient total CV‐related costs	461	474
Outpatient pharmacy CV‐related costs	66	67
Overall total non‐CV‐related costs	6844	7147
Inpatient total non‐CV‐related costs	2368	2493
Outpatient total non‐CV‐related costs	1474	1527
Outpatient pharmacy non‐CV‐related costs	3002	3126
Medication persistence, n (%)^d^		
P2Y12 inhibitor	13 151/15 391 (85.4)	19 548/22 786 (85.8)
Statin	23 234/28 953 (80.2)	29 036/36 101 (80.4)
ACE inhibitor	13 886/16 706 (83.1)	17 300/20 700 (83.6)
ARB	8659/10 334 (83.8)	10 920/12 987 (84.1)
β‐blocker	23 657/28 949 (81.7)	29 450/35 959 (81.9)

Abbreviations: ACE, angiotensin‐converting enzyme; ARB, angiotensin receptor blocker; CAD, coronary artery disease; CV, cardiovascular; IQR, interquartile range; SD, standard deviation; T2D, type 2 diabetes; THEMIS, Effect of Ticagrelor on Health Outcomes in Diabetes Mellitus Patients Intervention Study.

^a^ Fee‐for‐service patients only; patients without a claim in each relevant category were excluded.

^b^ Only includes costs for patients who experienced events during follow‐up.

^c^ Calculated by dividing the total costs over the study period by the mean duration of follow‐up.

^d^ Defined as continuation of a medication class over the study period without more than a 60‐day gap in medication supply after the last fill, as determined by the date and days’ supply dispensed. Denominators include patients with at least one claim for a given medication.

## 4 DISCUSSION

The findings from this retrospective, observational, cohort study showed substantial rates of death, nonfatal MI and nonfatal stroke, as well as substantial HRU and healthcare costs, in DCR patients who were similar to those who would have been eligible for THEMIS. Event rates and use of CV prevention medications were similar to patients in a broader cohort with T2D and CAD, some of whom would not have met THEMIS eligibility criteria. These findings indicate that the THEMIS trial population represents a group of higher‐risk patients among those with CAD and concomitant T2D. However, it should be noted that up to a fifth of patients in the ATHENA cohorts were not persistent with medications for CV prevention, which may have also contributed to high CV event rates.

Although the presence of T2D is a known risk factor for CV disease, there is a large gradient of CV risk among patients with T2D.^16^ Broadly speaking, patients can be categorized as having T2D and other CV risk factors but no established CAD; having established CAD but no prior ischaemic events (nonfatal MI or stroke); and having established CAD and prior ischaemic events. The incremental increase in the CV risk of patients in these populations was documented in a prospective observational study of patients in the Reduction of Atherothrombosis for Continued Health (REACH) registry. Here, the 4‐year cumulative incidence of CV death, nonfatal MI or nonfatal stroke was significantly higher in patients with diabetes and known atherothrombosis compared to those with diabetes and atherothrombotic risk factors alone (19.5% vs 9.5%; *P* < .001).^6^ Among the group of patients with diabetes and known atherothrombosis, the risk of the composite outcome was only slightly lower in those who had not experienced a prior ischaemic event compared with patients who had experienced an event previously.^6^

Studies attempting to address the question of whether antiplatelet therapies reduce the incidence of ischaemic events in patients with T2D have assessed groups of patients with T2D at both extremes of the CV risk continuum. In a recent randomized trial, A Study of Cardiovascular Events iN Diabetes (ASCEND), the absolute benefits of ASA, when administered at a dose of 100 mg/d, were largely offset by major bleeding events in patients with T2D and no evident CV disease.^17^ However, as the study investigators did not differentiate between the different types of bleeding, it was argued that many of the major bleeding events recorded were from gastrointestinal sources that could be mitigated with use of proton pump inhibitors. Similarly, in the Aspirin to Reduce Risk of Initial Vascular Events (ARRIVE) trial, ASA was not associated with a reduction in adverse CV events in patients with T2D who were deemed to have a moderate risk of experiencing a first CV event. Notably, the CV event rate was low in this study, suggesting that the patients were more representative of a low‐risk population.^18^

Conversely, dual antiplatelet regimens that are more potent than ASA alone have demonstrated a clear benefit in patients with T2D and a history of MI. In the Prevention of Cardiovascular Events in Patients With Prior Heart Attack Using Ticagrelor Compared to Placebo on a Background of Aspirin‐Thrombolysis In Myocardial Infarction 54 (PEGASUS‐TIMI 54) trial, patients with T2D had a greater absolute reduction in the risk of major adverse CV events than patients without diabetes when treated with a combination of ticagrelor and ASA (1.5% vs 1.1%).^19^ Moreover, a post hoc analysis of data from the Clopidogrel vs Aspirin in Patients at Risk of Ischemic Events (CAPRIE) trial suggested that patients with diabetes derived a greater benefit from clopidogrel than from ASA therapy.^20^

As a consequence of these trial findings, current clinical guidelines recommend the use of antiplatelet therapies in patients with T2D and prior MI or stroke, but not in those with low CV risk.^3^, ^8^ In particular, the 2019 guidelines from the American College of Cardiology (ACC) and the American Heart Association (AHA) advise against the use of ASA for the routine primary prevention of atherosclerotic CV disease, due to lack of net benefit in clinical trials.^10^ However, guidelines are less clear on their recommendations for the use of antiplatelet therapies in patients with T2D and established CV disease who have not experienced a prior ischaemic event. Therefore, THEMIS was designed to address the question of whether intensification of antiplatelet therapy beyond ASA reduces the risk of ischaemic events in this group of patients^11^ and showed that the incidence of ischaemic CV events was lower in patients receiving ASA and ticagrelor than in those receiving ASA and placebo (HR: 0.90, 95% CI: 0.81‐0.99, *P* = .04). However, the incidence of major bleeding events was significantly higher in the ticagrelor group than in the placebo group (HR: 2.32, 95% CI: 1.82‐1.94, *P* < .001).^12^

In the present study, rates of ischaemic events during follow‐up were high in both the THEMIS‐like and the broader T2D‐CAD cohorts. One‐third of patients with an initial MI or stroke in both cohorts experienced recurrent events. In particular, the rates of the composite outcome (16.34 [95% CI: 16.31‐16.37] and 17.64 (95% CI: 17.61‐17.67] per 100 person‐years for the THEMIS‐like cohort and CAD‐T2D cohort, respectively) were in line with the rate of recurrent CV events reported in a study of patients with T2D in a population‐based cohort of patients with CV disease (12.4 [95% CI: 8.5‐17.6]).^21^ The proportions of patients receiving CV prevention medications (statins/lipid‐lowering drugs, ACE inhibitors, ARBs and β‐blockers) at baseline were similar in the THEMIS study (89.8%, 42.4%, 37.5% and 73.8%, respectively)^11^ and our THEMIS‐like cohort (82.3%, 49.9%, 30.2% and 78.8%, respectively), suggesting similar levels of care in the clinical trial and our real‐world cohorts. However, the Kaplan‐Meier cumulative incidence at 2 years for all‐cause death, MI, stroke and the composite endpoint in the ATHENA study were approximately double those observed at 3 years in the THEMIS trial.^12^ This higher cumulative incidence of events over a shorter period of time may be explained, at least in part, by the fact that the ATHENA population was older than the THEMIS population (median age: 73 vs 66 years). Nevertheless, the cumulative incidence of the composite endpoint at 2 years in both ATHENA cohorts was slightly higher than those observed in patients aged >75 years in THEMIS after 3 years of follow‐up (13.6% and 13.1% in the ticagrelor and placebo group, respectively). Also, in the ATHENA subgroups of patients with a history of PCI or CABG, the event rates for the composite endpoint were higher than in the overall cohorts. Of note, in THEMIS, the subgroup of patients with a history of PCI experienced more pronounced benefit from ticagrelor than the overall THEMIS population.^13^

Our findings concur with the results from 4 years of follow‐up of patients with either established atherosclerosis or significant risk factors for atherosclerosis in the REACH registry, which reported 4‐year, age‐ and sex‐adjusted hazard rates of 14.3% (95% CI: 13.8‐14.9) for all‐cause death, 4.4% (95% CI: 4.0‐4.7) for nonfatal MI and 5.7% (95% CI: 5.3‐6.0) for nonfatal stroke, among patients with diabetes.^6^ The high rates of all‐cause death in the aforementioned study, as well as in the present study, are notable and likely attributable to the enrolment of elderly patients with multiple comorbidities. The rates of all‐cause death, MI and ischaemic stroke (1.6, 1.3 and 0.7 events per 100 person‐years, respectively) were notably much lower in placebo‐treated patients with T2D in the Dapagliflozin Effect on Cardiovascular Events–Thrombolysis in Myocardial Infarction 58 (DECLARE‐TIMI 58) randomized trial than in the present study, which highlights the discrepancy between event rates in real‐world studies and randomized trials. However, a recent and comparable database study in a general US population of patients with CAD, with and without diabetes (N = 85 754; 31.8% of patients with diabetes), reported lower incidence rates of MI and stroke (1.95 and 1.80 events per 100 person‐years for MI and stroke, respectively)^22^ than those observed in ATHENA, thus suggesting that T2D is associated with an increased CV risk in patients with CAD.

The costs associated with treating patients in both the THEMIS‐like and CAD‐T2D cohorts were substantial, and a large proportion was attributable to inpatient stays. These findings concur with those from a 6‐year, longitudinal analysis of claims data gathered from patients newly diagnosed with T2D in the USA, which also observed an increase in total healthcare costs of 33% over the study period.^23^ In the present study of primarily elderly patients with multiple comorbidities, approximately one‐fifth of all costs were CV‐related, which emphasizes the high burden of CV complications in patients with T2D. In particular, the costs of hospitalization for MI and stroke accounted for approximately one‐quarter of inpatient CV‐related costs in both cohorts. Patients in both cohorts had a lower risk of bleeding events (0.13 events per 100 person‐years) than those observed in primary prevention trials of ASA, despite the majority being treated with antiplatelet therapies (ASA alone and dual antiplatelet therapies) and the relatively high mean age of patients in both cohorts. In ASCEND, a placebo‐controlled study of ASA for the prevention of CAD in patients with T2D, the incidence rates of major bleeding events in patients with T2D taking ASA were 0.36, 0.57 and 1.09 per 100 person‐years for those at low, medium and high CV risk, respectively.^17^ Although it was difficult to measure persistence with OAP therapies accurately because the over‐the‐counter use of ASA was not recorded, it should be noted that OAP persistence appeared to be low in both ATHENA cohorts, which may have contributed to the small number of bleeding events.

Notable strengths of the present study include the use of real‐world data from a clinical practice‐based registry linked to a comprehensive administrative claims database, the large sample size and US nationwide scope of the data. The study criteria were closely aligned with eligibility criteria for THEMIS, in order to inform the generalizability of the trial results to clinical practice. Limitations include missing data and the potential for misclassification bias due to coding errors. This precluded the stratification of our findings by severity of T2D and presence of comorbidities such as chronic kidney disease because key variables, including glycated haemoglobin and estimated glomerular filtration rate, were missing in large proportions of patients. As with any similar database, representativeness to the general population of patients with T2D in the USA cannot be assumed. In particular, ethnicity was not recorded for more than a quarter of DCR patients included in the THEMIS‐like and T2D‐CAD cohorts. As such, the representativeness of the ethnic distribution of these cohorts could not be assessed. In addition, ATHENA included patients enrolled in Medicare, who were mostly 65 or older; results from the study may not be generalizable to younger populations. The DCR is a voluntary, practice‐based registry in which the majority of patients are cared for by specialist physicians; thus, the prevalence of comorbidities may be higher than in cohorts from nationally representative data sets, such as those from the National Health And Nutrition Examination Survey (NHANES).^24^ Indeed, the DCR was highly enriched with cardiology practices during the years for which data were included. The high level of care of patients in the present analysis also gives rise to the possibility that the frequency of events and outcomes are lower than would be seen in comparable populations from other US data sources. For example, more than 80% of patients were using lipid‐lowering medications, and median low‐density lipoprotein cholesterol levels were close to those recommended by clinical guidelines for patients with diabetes and atherosclerotic CV disease. The study follow‐up period was also relatively brief because linked data were only available for a 2‐year period (from 1 January 2013 to 31 December 2014), so findings may change with longer follow‐up. However, the findings from this analysis provide important preliminary data from comparable clinical practice‐based cohorts in the USA to complement findings from THEMIS.

In conclusion, findings from this study demonstrated substantial rates of ischaemic events and all‐cause death in patients with CAD and concomitant T2D, accompanied by considerable healthcare utilization and associated costs. This suggests a potential opportunity for improved management of these patients, which may include better persistence with CV prevention medications, better adherence to clinical guidelines and treatment with long‐term dual antiplatelet therapy, to improve outcomes in this high‐risk population.

## CONFLICT OF INTEREST

Eric Wittbrodt, Narinder Bhalla, Karolina Andersson Sundell, Phillip Hunt and Carl Mellström are employees of AstraZeneca (significant relationships). Qi Gao and Liyan Dong are employees of the Baim Institute for Clinical Research (significant relationships). Nathan Wong is a consultant for AstraZeneca (modest relationship). Matthew Cavender has received research support (nonsalary) from AstraZeneca, Bristol‐Myers Squibb, Chiesi, GlaxoSmithKline, Novartis, Takeda and The Medicines Company, and research support with salary from Novo Nordisk; he has also received modest consulting fees from AstraZeneca, Boehringer Ingelheim, Chiesi, Edwards Lifesciences, Janssen, Merck and Sanofi‐Aventis.

## AUTHORS’ CONTRIBUTIONS

All authors contributed to the design of the analysis. Qi Gao and Liyan Dong conducted the statistical analysis. Eric Wittbrodt and Phillip Hunt developed the first draft of the manuscript, which was critically reviewed by all authors for important intellectual content. The final version of the manuscript was approved by all authors before submission. Eric Wittbrodt is the guarantor of this work.

## ETHICAL APPROVAL

A waiver of written informed consent and authorization for this study were granted by Chesapeake Research Review, Inc., because DCR participation does not require data collection beyond that of routine clinical care and data are de‐identified.

## Supporting information

Table S1

## Data Availability

Data underlying the findings described in this manuscript may be obtained in accordance with AstraZeneca's data sharing policy described at https://astrazenecagroup‐dt.pharmacm.com//DT/Home/Index/.
