# Supplementary material for: Assessment of the high risk and unmet need in patients with CAD and type 2 diabetes (ATHENA): US healthcare resource utilization, cost and burden of illness in the Diabetes Collaborative Registry
Source: Endocrinol Diabetes Metab. 2020 May 7;3(3):e00133. doi: 10.1002/edm2.133 (PMC7375123; doi:10.1002/edm2.133)
Supplement: Supplementary file 1 — Table S1 [file EDM2-3-e00133-s001.docx]

# Supplemental material

**Supplementary Table 1. Kaplan–Meier estimates for the cumulative incidence of clinical outcomes in the THEMIS-like and CAD-T2D cohorts during follow-up**

| **Follow-up timepoint, days** | **0** | **90** | **180** | **270** | **360** | **450** | **540** | **630** | **720** |
| --- | --- | --- | --- | --- | --- | --- | --- | --- | --- |
| **Composite outcome** | | | | | | | | | |
| **THEMIS-like cohort** |  |  |  |  |  |  |  |  |  |
| Entered, n | 56,040 | 55,973 | 51,194 | 46,291 | 40,897 | 34,675 | 29.104 | 22,567 | 12,977 |
| Censored, n | 38 | 3417 | 3897 | 4567 | 5432 | 4927 | 6025 | 9195 | 11,559 |
| Events, n | 29 | 1362 | 1006 | 827 | 790 | 644 | 512 | 395 | 175 |
| Cumulative incidence, % | 0.1 | 2.6 | 4.6 | 6.3 | 8.3 | 10.1 | 11.9 | 13.8 | 15.9 |
| Greenwood SE, % | 0.0 | 0.1 | 0.1 | 0.1 | 0.1 | 0.1 | 0.2 | 0.2 | 0.3 |
| **CAD-T2D cohort** |  |  |  |  |  |  |  |  |  |
| Entered, n | 69,790 | 69,692 | 63,197 | 56,576 | 49,719 | 41,845 | 35,030 | 27,124 | 15,603 |
| Censored, n | 53 | 4591 | 5279 | 5801 | 6889 | 6034 | 7267 | 11,031 | 13,902 |
| Events, n | 45 | 1904 | 1342 | 1056 | 985 | 781 | 639 | 490 | 205 |
| Cumulative incidence, % | 0.1 | 2.9 | 5.0 | 6.9 | 8.9 | 10.7 | 12.5 | 14.5 | 16.4 |
| Greenwood SE, % | 0.0 | 0.1 | 0.1 | 0.1 | 0.1 | 0.1 | 0.1 | 0.2 | 0.2 |
| **All-cause death** | | | | | | | | | |
| **THEMIS-like cohort** |  |  |  |  |  |  |  |  |  |
| Entered, n | 56,040 | 56,000 | 51,973 | 47,408 | 42,175 | 36,013 | 30,422 | 23,768 | 13,801 |
| Censored, n | 38 | 3461 | 3985 | 4720 | 5653 | 5148 | 6298 | 9675 | 12351 |
| Events, n | 2 | 566 | 580 | 513 | 509 | 443 | 356 | 292 | 125 |
| Cumulative incidence, % | 0.0 | 1.0 | 2.2 | 3.3 | 4.6 | 5.8 | 7.0 | 8.4 | 10.0 |
| Greenwood SE, % | 0.0 | 0.0 | 0.1 | 0.1 | 0.1 | 0.1 | 0.1 | 0.2 | 0.2 |
| **CAD-T2D cohort** |  |  |  |  |  |  |  |  |  |
| Entered, n | 69,790 | 69,735 | 64,333 | 58,126 | 51,441 | 43,606 | 36,739 | 28,658 | 16,660 |
| Censored, n | 53 | 4666 | 5427 | 6032 | 7210 | 6329 | 7637 | 11,646 | 14,918 |
| Events, n | 2 | 736 | 780 | 653 | 625 | 538 | 444 | 352 | 143 |
| Cumulative incidence, % | 0.0 | 1.1 | 2.3 | 3.5 | 4.8 | 6.0 | 7.3 | 8.7 | 10.1 |
| Greenwood SE, % | 0.0 | 0.0 | 0.1 | 0.1 | 0.1 | 0.1 | 0.1 | 0.1 | 0.2 |

| **Non-fatal myocardial infarction** | | | | | | | | | |
| --- | --- | --- | --- | --- | --- | --- | --- | --- | --- |
| **THEMIS-like cohort** |  |  |  |  |  |  |  |  |  |
| Entered, n | 56,040 | 55,996 | 51,526 | 46,787 | 41,472 | 35,260 | 29,671 | 23,096 | 13,341 |
| Censored, n | 40 | 3940 | 4441 | 5083 | 5982 | 5412 | 6424 | 9649 | 12,006 |
| Events, n | 4 | 530 | 298 | 232 | 230 | 177 | 151 | 106 | 53 |
| Cumulative incidence, % | 0.0 | 1.0 | 1.6 | 2.1 | 2.7 | 3.2 | 3.8 | 4.3 | 5.0 |
| Greenwood SE, % | 0.0 | 0.0 | 0.1 | 0.1 | 0.1 | 0.1 | 0.1 | 0.1 | 0.1 |
| **CAD-T2D cohort** |  |  |  |  |  |  |  |  |  |
| Entered, n | 69,790 | 69,728 | 63,723 | 57,306 | 50,533 | 42,655 | 35,809 | 27,829 | 16,094 |
| Censored, n | 55 | 5271 | 6032 | 6478 | 7585 | 6639 | 7799 | 11,600 | 14,484 |
| Events, n | 7 | 734 | 385 | 295 | 293 | 207 | 181 | 135 | 59 |
| Cumulative incidence, % | 0.0 | 1.1 | 1.7 | 2.3 | 2.9 | 3.4 | 3.9 | 4.5 | 5.1 |
| Greenwood SE, % | 0.0 | 0.0 | 0.1 | 0.1 | 0.1 | 0.1 | 0.1 | 0.1 | 0.1 |
| **Non-fatal ischemic stroke** | | | | | | | | | |
| **THEMIS-like cohort** |  |  |  |  |  |  |  |  |  |
| Entered, n | 56,040 | 55,977 | 51,647 | 46,915 | 41,606 | 35,420 | 29,847 | 23,236 | 13,431 |
| Censored, n | 40 | 3979 | 4500 | 5115 | 6016 | 5437 | 6507 | 9721 | 12,114 |
| Events, n | 23 | 351 | 232 | 194 | 170 | 136 | 104 | 84 | 30 |
| Cumulative incidence, % | 0.0 | 0.7 | 1.2 | 1.6 | 2.0 | 2.4 | 2.8 | 3.2 | 3.6 |
| Greenwood SE, % | 0.0 | 0.0 | 0.0 | 0.1 | 0.1 | 0.1 | 0.1 | 0.1 | 0.1 |
| **CAD-T2D cohort** |  |  |  |  |  |  |  |  |  |
| Entered, n | 69,790 | 69,699 | 63,818 | 57,400 | 50,631 | 42,784 | 35,949 | 27,944 | 16,156 |
| Censored, n | 55 | 5328 | 6087 | 6510 | 7625 | 6657 | 7875 | 11,679 | 14,570 |
| Events, n | 36 | 553 | 331 | 259 | 222 | 178 | 130 | 109 | 40 |
| Cumulative incidence, % | 0.1 | 0.9 | 1.4 | 1.9 | 2.3 | 2.8 | 3.2 | 3.6 | 4.0 |
| Greenwood SE, % | 0.0 | 0.0 | 0.0 | 0.1 | 0.1 | 0.1 | 0.1 | 0.1 | 0.1 |
| **Non-fatal stroke** | | | | | | | | | |
| **THEMIS-like cohort** |  |  |  |  |  |  |  |  |  |
| Entered, n | 56,040 | 55,977 | 51,622 | 46,878 | 41,559 | 35,374 | 29,796 | 23,193 | 13,408 |
| Censored, n | 40 | 3975 | 4492 | 5105 | 6003 | 5421 | 6492 | 9693 | 12,090 |
| Events, n | 23 | 380 | 252 | 214 | 182 | 157 | 111 | 92 | 37 |
| Cumulative incidence, % | 0.0 | 0.7 | 1.3 | 1.7 | 2.2 | 2.7 | 3.1 | 3.5 | 4.0 |
| Greenwood SE, % | 0.0 | 0.0 | 0.0 | 0.1 | 0.1 | 0.1 | 0.1 | 0.1 | 0.1 |
| **CAD-T2D cohort** |  |  |  |  |  |  |  |  |  |
| Entered, n | 69,790 | 69,699 | 63,783 | 57,351 | 50,575 | 42,726 | 35,884 | 27,892 | 16,125 |
| Censored, n | 55 | 5323 | 6073 | 6494 | 7610 | 6641 | 7854 | 11,648 | 14,540 |
| Events, n | 36 | 593 | 359 | 282 | 239 | 201 | 138 | 119 | 46 |
| Cumulative incidence, % | 0.1 | 0.9 | 1.5 | 2.0 | 2.5 | 3.0 | 3.4 | 4.0 | 4.4 |
| Greenwood SE, % | 0.0 | 0.0 | 0.0 | 0.1 | 0.1 | 0.1 | 0.1 | 0.1 | 0.1 |

CAD, coronary artery disease; SE, standard error; T2D, type 2 diabetes; THEMIS, Effect of Ticagrelor on Health Outcomes in Diabetes Mellitus Patients Intervention Study.
